# Study of different heterocycles showing significant anti-severe acute respiratory syndrome 2 activity *in vitro* and *in vivo*

**DOI:** 10.14202/vetworld.2024.1281-1290

**Published:** 2024-06-14

**Authors:** Aleksandr Yengoyan, Tiruhi Gomktsyan, Vergush Pivazyan, Emma Ghazaryan, Roza Shainova, Armen Karapetyan, Diana Avetyan, Levon Aslanyan, Karine Baroyan, Alexander Tuzikov, Mariam Sargsyan, Bagrat Baghdasaryan, Nane Bayramyan, Sona Hakobyan, Arpine Poghosyan, Aida Avetisyan, Hranush Avagyan, Lina Hakobyan, Karalyan Zaven

**Affiliations:** 1 Department of Pesticides Synthesis and Expertise National Agrarian University of Armenia, Teryan 74, Yerevan, 0009, Armenia; 2 Department of Chemistry Laboratory of Structural Bioinformatics, Russian-Armenian University, H. Emin, 123, Yerevan, 0051, Armenia; 3 Laboratory of Human Genomics and Immunomics, Institute of Molecular Biology, National Academy of Sciences RA, Yerevan, 0014, Armenia; 4 Department of Mathematics, Institute for Informatics and Automation Problems of NAS RA, Yerevan, Armenia; 5 Department of Anatomy, Yerevan State Medical University after M. Heratsi, Armenia Yerevan, Armenia; 6 United Institute of Informatics Problems, National Academy of Sciences of Belarus, Belarus; 7 Department of Epidemiology and Parasitology, Armenian National Agrarian University, Yerevan, Armenia; 8 Laboratory of Cell Biology and Virology, Institute of Molecular Biology, National Academy of Sciences RA, Yerevan, 0014, Armenia; 9 Department of Human Anatomy, Yerevan State Medical University after M. Heratsi, Yerevan, Armenia

**Keywords:** broad-spectrum antiviral agents, heterocycle compounds, *in vitro*, *in vivo*, severe acute respiratory syndrome-related coronavirus, Syrian hamsters

## Abstract

**Background and Aim:**

With the emergence of severe acute respiratory syndrome-related coronavirus (SARS-CoV-2), antiviral drug development has gained increased significance due to the high incidence and potentially severe complications of the resulting coronavirus infection. Heterocycle compounds, acting as antimetabolites of DNA and RNA monomers, rank among the most effective antiviral drugs. These compounds’ antiviral effects on various SARS-CoV-2 isolates, as found in existing data collections, form the basis for further research. The aim of this study was to examine the possible antiviral effect of some originally synthesized heterocyclic compounds.

**Materials and Methods:**

The main methods were cell culturing, cytotoxicity assay, qRT-PCR assay, tissue and blood cells analysis, and micro-computed tomography (micro-CT) imaging.

**Results:**

In both *in vitro* and *in vivo* conditions, the elimination of SARS-Cov-2 occurred significantly earlier after administration of the compounds compared to the control group. In hamsters, the primary symptoms of coronavirus disease disappeared following administration of heterocycle compounds.

**Conclusion:**

Using delta and omicron strains of the SARS-CoV-2 virus, newly created heterocycle compound analogs dramatically reduced SARS-CoV-2 multiplication, resulting in a drop in viral RNA load in the supernatant under in vitro conditions. Improvements in pathological manifestations in the blood, bone marrow, and internal organs of hamsters demonstrated that heterocycle compounds inhibited SARS-CoV-2 replication both *in vitro* and *in vivo*.

## Introduction

The importance of antiviral drug development is heightened due to the widespread severe acute respiratory syndrome-related coronavirus (SARS-CoV-2) infection, which has a high incidence rate and poses a risk of complications. Prioritizing the creation of broad-spectrum antiviral drugs or those specifically targeting SARS-CoV-2 is crucial [1, 2]. Given the low efficacy against new strains, many side effects, lack of absolute protection, and limited availability of vaccines, serve as a vital tool in combating SARS-CoV-2. Developing and modifying vaccines are a time-consuming, costly process involving extended periods of checks and analysis for validity [3]. The importance of antiviral drugs that inhibit SARS-CoV-2 replication and mitigate complications becomes evident.

Heterocyclic scaffolds, which have been explored for over half a century, possess anticancer, antimalarial, anti-inflammatory, antitubercular, antimicrobial, antidiabetic, antiviral, and other therapeutic functions [4]. They are vital for life, performing a pivotal function in cell metabolism. These molecules consist of RNA and DNA bases, amino acids, vitamins, pigments, hormones, and sugars. Several heterocyclic compounds used in medicine are antibiotics, alkaloids, and cardiac glycosides. In pharmaceuticals, synthetic heterocyclic functions as anticancer agents, analgesics, hypnotics, and vasopressor modifiers. They function as pesticides, insecticides, herbicides, and rodenticides. These compounds can be synthesized with novel structures and a broad spectrum of properties and reactivity due to their complexity [5]. The foundation for the study of this group of substances is, first of all, the numerous available data on the antiviral activities of these compounds on different isolates of SARS-CoV-2 [6–8].

Over the past two decades, there has been growing interest in fused bicyclic heterosystems with varying pyrimidine-pyridazine conjunctions and a 1,2,4-triazole ring. 1,2,4-triazolo[4,3-c] pyrimidine derivatives were found to exhibit anticonvulsant [9], anticancer [10], antimicrobial [11, 12], neurotropic [13], and antagonistic effects on adenosine receptors [14] properties. Among the fused [1,2,4] triazolo[4,3-b] pyridazines, novel compounds with antibacterial, antifungal, antihypertensive, anticonvulsant, and anxiolytic activities [14], phosphodiesterase-4 inhibitors [15], and gamma-aminobutyric acid receptor ligands including selective agonists for α2- and α3- subtypes were identified [16, 17]. Nucleotide analogs, acting as aberrant monomers of DNA and RNA, rank among the most effective antiviral drugs [1, 2].

The replication of viruses can be inhibited by heterocycle compounds, depending on the hydrophobic substituent’s structure and position. Our study aimed to explore the antiviral potential of original drugs featuring bicyclic heterosystems, specifically the arrangement of pyrimidine and pyridazine cycles around a 1,2,4-triazole ring.

## Material and Methods

### Ethics approval

Animal care and euthanasia were conducted adhering to the American Veterinary Medical Association (AVMA) and local guidelines (Institutional Review Board/Independent Ethics Committee of the Institute of Molecular Biology of NAS, approval number IRB00004079).

### Study period and location

The study was conducted from January to December 2022 at the Institute of Molecular Biology.

### Cells and the virus

The SARS-CoV-2 delta variant emerged from a coronavirus disease-19 patient’s nasal and oral mucosa [18] and multiplied in Vero and Vero E6 cells, which were cultivated in Eagle-Dulbecco’s Minimal Essential Media (Sigma Aldrich, USA) with additives of 10% heat-inactivated fetal bovine serum (Sigma Aldrich), 2 mM L-glutamine, and 1 mM sodium pyruvate. 2 × 10^5^ cells/mL was used to seed the cells. In an enhanced BSL3 laboratory, all SARS-CoV-2 experiments were carried out. 0.1 TCD50/mL of SARS-CoV-2 virus was used to infect cells. The cells were incubated at 37°C under 5% CO_2_ conditions. At –80°C, the supernatants of cell cultures infected for 1–7 days were collected, filtered, and stored.

96-well cell culture plates containing confluent cells at a density of 1 × 10^4^ cell/well were used for titrating SARS-CoV-2 from thawed samples. The final examination for cytopathic effect (CPE) in virus doses was conducted after 9 days of daily checks. The Reed and Muench method was used to determine the virus titer. Uninfected cells filled the wells for control experiment. The virus titer was given as TCD50/mL.

We determined CC50 values for each investigated chemical: 5a - 240 μg/mL, 5b - 100 μg/mL, 5c - 40 μg/mL, 5d - 320 μg/mL, 6a - 20 μg/mL, 6d - 320 μg/mL, 8 - 480 μg/mL, 10a - 40 μg/mL, 10b - 240 μg/mL, 10d - 480 μg cell viability/CPE reduction assay and quantitative reverse transcription polymerase chain reaction (qRT-PCR) was employed for identifying antiviral substances against SARS-CoV-2.

### Cytotoxicity

The neutral red uptake test was used to evaluate the cytotoxicity of the tested compounds [19]. 96-well plate containing Confluent Vero E6 cells at 1 × 10^4^ cells/well was treated with escalating doses of the target heterocycle compounds. The treated cells were cultured at 37°C under 5% CO_2_ for 72 h.

### qRT-PCR assay

The SARS-CoV-2 viral load in Vero cells and Syrian hamster samples was determined by isolating viral RNA with theHiGene™ Viral RNA/DNA Prep Kit (Biofact, Yuseong-gu, Darjeon, Republic of Korea), according to the manufacturer’s instructions. The FIREScript RT cDNA synthesis kit (Solis Biodyne, Teaduspargi 9, Tartu, Estonia) was used to reverse transcribe isolated RNA from the samples. A standard curve on an Eco Illumina real-time polymerase chain reaction system (Illumin Inc, San Diego, CA, USA) was used to perform real-time qRT-PCR [20]. The reaction mixtures were prepared according to the kit instructions for the HOT FIREPol EvaGreen qRT-PCR mix plus (Solis Biodyne San Diego, California, USA). Standard curves were produced with a 10-fold dilution sequence. The Ct value was determined by the ECO-Illumina system software San Diego, California [Eco Software v5.0 (illumina.com)]. In this study, we used S gene primers, Fwd: CCTACTAAATTAAATGATCTCTGCTTTACT and Rev: CAA GCT ATA ACG CAG CCT GTA as desccribed by Pearson *et al*. [21].

### Experimental infection of Syrian hamsters

Forty-two 7–9-month-old male Syrian hamsters were used for the experiments. Body weights were measured before infection. At a BSL-3 facility, animals were provided unlimited supplies of food and water. Hamsters received anesthesia through intraperitoneal injection of ketamine-xylazine. They were infected with 106 TCD50/mL (in 110 μL) SARS-CoV-2 through intranasal (100 μL) and ocular (10 μL) routes. Seven hamsters served as the control group. For 14 consecutive days, both body weight and temperature were monitored daily. For daily blood smear examinations, blood from the gingival vein was utilized [22, 23].

All hamsters were divided into 6 groups (7 animals in each group): 1^st^ - control group; 2^nd^ - NA #5d; 3^rd^ - NA #8; 4^th^ - NA #10b; 5^th^ - NA #10d; and 6^th^ -remdesivir.

Remdesivir (Cipla) was administered intraperitoneally (i.p.) as an aqueous solution at a dose of 15 mg/kg.

In acute toxicity studies, drug doses were used that were 5 times higher than the therapeutic dose (number of hamsters: 24) (Table-S1, supplementary data).

**Table S1:** Acute toxicity data of investigated preparations on hamsters.

Groups	Animal number	Dose mg/kg	Number of dead hamsters after 2 days	Number of dead hamsters after 7 days
#5d	3	0.2	0	0
	3	1.0	0	0
#8	3	0.3	0	0
	3	1.5	0	0
#10b	3	0.16	0	0
	3	0.8	0	1*
#10d	3	0.32	0	0
	3	1.6	0	0

* Autopsy and histological studies revealed massive hemorrhages in the abdominal cavity and leukopenia. In all investigated preparations except 10b, no lethal effect was observed throughout the experiment. No abnormal appearance or behavior was noted in all investigated groups.

### Micro-computed tomography (micro-CT) imaging

The Bruker SkyScan 1276 micro-CT system (Bruker, Karlsruhe, Germany)was employed to examine the histology of hamsters’ lungs previously infected and treated with heterocycle compounds. Hamsters underwent free-breathing scans. An anesthetic was administered using ketamine-xylazine [24].

### Tissue samples, blood smears, bone marrow imprints, and cell analysis

On days 5^th^, 7^th^, 8^th^, and 18^th^ post-infection, eight hamsters were euthanized using sodium thiopental and their organs (lungs, liver, kidney, bone marrow, lymphatic nodes, spleen, and blood) were subsequently collected. At the Institute of Molecular Biology, Syrian hamsters infected with SARS-CoV-2 were studied in Animal Biosafety Level 3 facilities. Slides for blood cell analysis were fixed in methanol and stained using the Pappenheim method, following the manufacturer’s instructions from Sigma-Aldrich. One hundred randomly chosen fields, each measuring 0.01 mm^2^, were analyzed and counted under a light microscope (Microm HM 355, Thermo Fisher Scientific, Waltham, MA USA) at 1250× magnification. Organ samples were fixed in 10% buffered formalin and stained with hematoxylin and eosin (Sigma-Aldrich). The histological examination was conducted with a light microscope [25].

### Statistical analysis

Each *in vitro* experiment was repeated three times. Significance was assessed using two-tailed student’s t-test and Mann–Whitney U-test, with p < 0.05 being considered significant; the Statistical Package for the Social Sciences version 17.0 (IBM Corp., NY, USA) was employed for analysis.

## Results

### Chemistry

**Scheme-1:**
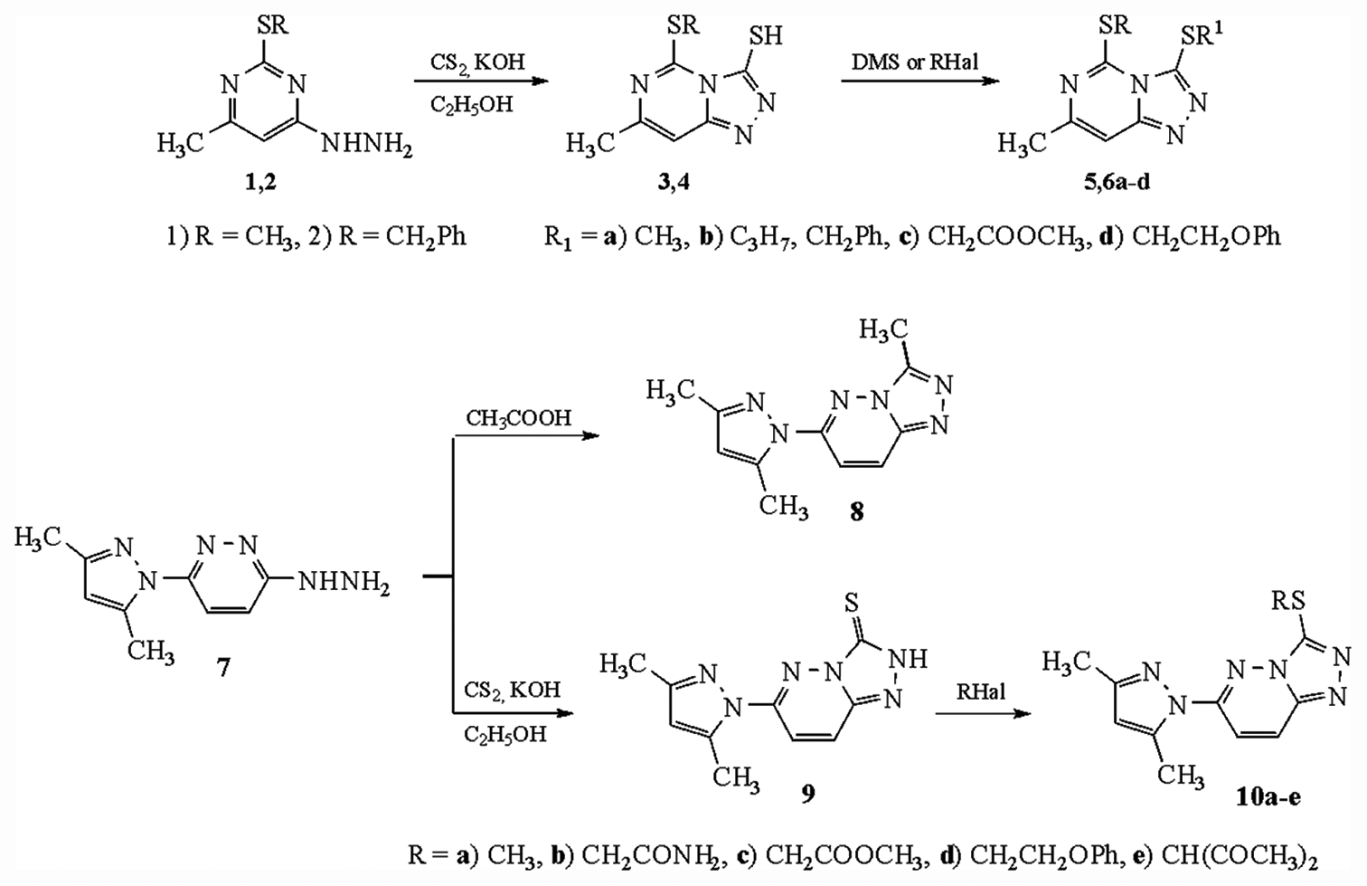
Synthesis of triazolopyrimidine and triazolopyridazine derivatives.

The Scheme-1 synthesized the target derivatives of fused bicyclic [1,2,4] triazolo [4,3-c] pyrimidine and [1,2,4] triazolo [4,3-b] pyridazine. These compounds’ physicochemical and spectral parameters align with previous findings [26, 27].

### Evaluation of toxicity of heterocycle compounds under *in vitro* conditions

96-well plates were utilized for VERO cell cultivation, with conditions set at 5% CO_2_ saturation and 100% humidity. 0.8/0.2 μm Gelman Sciences filters (Merck KGaA, Darmstadt, Germany) were used to sterilize heterocycle compound samples before their addition to culture plates containing VERO cells. The monolayer destruction degree in VERO cells was assessed as a measure of heterocycle compounds’ toxicity. The selected drug concentrations for antiviral testing, as determined by toxicity analysis, are 5a - 60 μg/mL, 5b - 25 μg/mL, 5c - 10 μg/mL, 5d - 80 μg/mL, 6a - 5 μg/mL, 6d - 80 μg/mL, 8 - 120 μg/mL, 10a - 10 μg/mL, 10b - 60 μg/mL, and 10d - 120 μg/mL.

**Figure-1:**
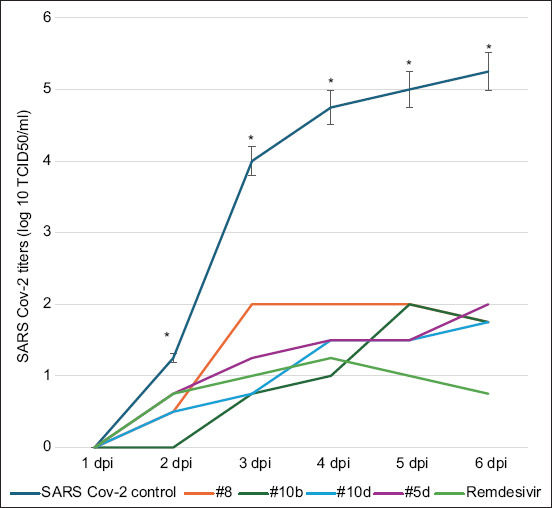
Suppression of severe acute respiratory syndrome-related coronavirus replication in VERO cells by heterocycle compounds. A significant difference is observed compared with the corresponded titers from groups treated with heterocycle compounds for 2-day post-infection (p < 0.01). The standard deviation in the titers of viruses under the action of heterocycle compounds is not shown since they overlap each other (however, their maximum values do not exceed 10%).

1 h incubation of Vero cells with a 100 TCD50/mL dose of an adapted SARS-CoV-2 isolate was used to assess its AV activity. The cells were washed with PBS to remove the unbound virus. This procedure was followed by the addition of all preparations. 24–224 h post-infection, the supernatant was collected and titrated by CPE-based assay every day. The studied heterocycle compounds (N 5d, 8, 10b, 10d) inhibited viral activity for 24–72 h, as shown by the study. Up to a dilution of 100 TCD50/ml (Figure-1), the viral activity was almost completely suppressed (Table 1).

**Table-1:** Evaluation of the toxicity on VERO cells under *in vitro* conditions.

Prep. (#)	Concentrations of primary solutions (mg/5mL DMCO)	Time of incubation (h)	Dilutions of primary solutions (%)									

			6.25	3.1	1.6	0.8	0.4	0.2	0.1	0.05	0.025	0.01
5a	150	24	-	-	-	+	+++	+++	+++	+++	+++	+++
		48	-	-	-	±	+++	+++	+++	+++	+++	+++
5b	250	24	-	-	-	-	-	-	++	+++	+++	+++
		48	-	-	-	-	-	-	++	+++	+++	+++
5c	100	24	-	-	-	-	-	-	++	+++	+++	+++
		48	-	-	-	-	-	-	+	+++	+++	+++
5d	100	24	-	-	+	+++	+++	+++	+++	+++	+++	+++
		48	-	-	+	+++	+++	+++	+++	+++	+++	+++
6a	50	24	-	-	-	-	-	-	+	+++	+++	+++
		48	-	-	-	-	-	-	±	+++	+++	+++
6d	200	24	-	-	+	+++	++	+++	+++	+++	+++	+++
		48	-	-	+	+++	+	++	+++	+++	+++	+++
8	150	24	-	-	-	++	+++	+++	+++	+++	+++	+++
		48	-	-	-	±	+++	+++	+++	+++	+++	+++
10a	40	24	-	-	-	-	++	+++	+++	+++	+++	+++
		48	-	-	-	-	+	++	+++	+++	+++	+++
10b	80	24	-	-	-	+++	+++	+++	+++	+++	+++	+++
		48	-	-	-	+++	+++	+++	+++	+++	+++	+++
10d	160	24	-	-	-	+++	+++	+++	+++	+++	+++	+++
		48	-	-	-	++	+++	+++	+++	+++	+++	+++

Evaluation of the toxicity on VERO cells under *in vitro* conditions. The arbitrary designations present the values of how much the monolayer maintained or preserved under the influence of our compounds. -=Monolayer not maintained, ±=Monolayer not maintained, normal cells were noticed, +=Monolayer maintained, ++=Monolayer maintained very well, +++=Monolayer changes were not detected compared to the control

We tested the *in vivo* antiviral activity of compounds NN 5d, 8, 10b, and 10d against SARS-CoV-2 in hamsters following infection through the intranasal and ocular routes. For initial research and dose selection *in vivo* trials, VERO cell lines were employed in *in vitro* studies.

### Clinical features of coronavirus infection in hamsters

**Figure-2:**
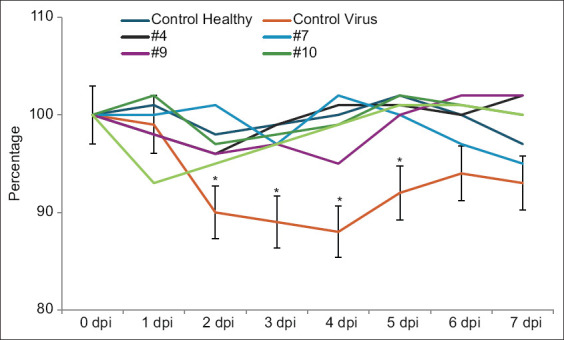
Dynamics of the body weight in severe acute respiratory syndrome-related coronavirus-infected hamsters and under the treatment by heterocycle compounds. *Significant compared with corresponded titers from groups treated with heterocycle compounds (p < 0.05, p < 0.01).

In the absence of any treatment, animals in the control-infected group lost 10–14% of their weight within the 1^st^-week post-SARS-CoV-2 infection. Intramuscularly administered groups did not exhibit body weight loss as shown in Figure-2. The standard deviations of body weight in the animal groups treated with heterocycle compound analogs overlap and are below a 10% variation.

### Lung pathology in experimental coronavirus infection

In all infected animals, except the controls, micro-CT analysis exposed serious lung abnormalities. 32–48 h post-virus inoculation, Syrian hamsters suffer lung damage. Computed tomography produces distinct images of internal organs. After 2 days of infection, all animals underwent CT scans to record signs of viral pneumonia in hamsters from all groups. 2 days after infection, the patient showed signs of lung abnormalities, appearing as fuzzy, patchy, “ground-glass” opacity. This lesion represents standard lung tissue damage.

**Figure-3:**
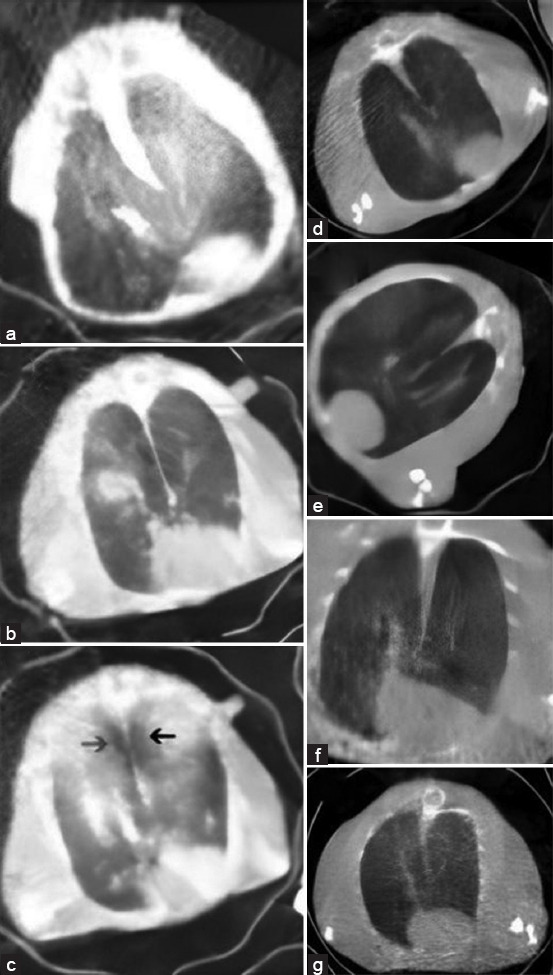
Lung pathology in hamsters after infection by severe acute respiratory syndrome-related coronavirus (SARS Cov-2) (delta strain). (a) Control SARS Cov-2 hamster group 5-day post-infection. (b) Control SARS-Cov-2 hamster group 7-day post-infection (dpi). (c) Control SARS-Cov-2 hamster group 7 dpi noticeable emphysema (arrowed). (d) SARS-Cov-2-infected hamsters after treatment with preparation #10b; 5 dpi. (e) SARS-Cov-2-infected hamsters after treatment with preparation #10d; 5 dpi. (f) SARS-Cov-2-infected hamsters after treatment with preparation #5d; 5 dpi. (g) SARS-Cov-2-infected hamsters after treatment with preparation #8; 5 dpi.

35% of the lung volume was affected in all hamsters by the 2^nd^-day post-infection (dpi), as shown in Figure-3a-c. The symptoms disappeared completely around 10–15 dpi, while the affected lungs decreased to about 25% of their original volume by the 5–6^th^ day. In Syrian golden hamsters with infections, the initial pathological manifestations were marked by local ground-glass opacities arranged peribronchially (Figure-3a). At 4–5 dpi, lung abnormalities worsened, featuring peripherally located, rounded, ground-glass opacities, and focal consolidations (Figure-3b). From 5 to 9 dpi, the most severe lung damage ensued with extensive tissue damage, micropulmonary rupture, and local emphysema formation (Figure-3c). 7–10 dpi, lung regeneration occurred in certain animals, with gradually decreasing ground-glass opacities; residual minimal abnormalities were detected at 12 dpi. 5-day post-injury, the development of all types of lung injury is inhibited by NA NN 5d, 8, 10b, 10d. 3D, E, F, and G are the elements in question. Preparations can hinder the progression of serious lung disorders such as extensive tissue damage, micropulmonary rupture, and local emphysema formation (Figure-3d-g).

### Viral load in washes of the mucous membranes of the nose and mouth of hamsters

**Figure-4:**
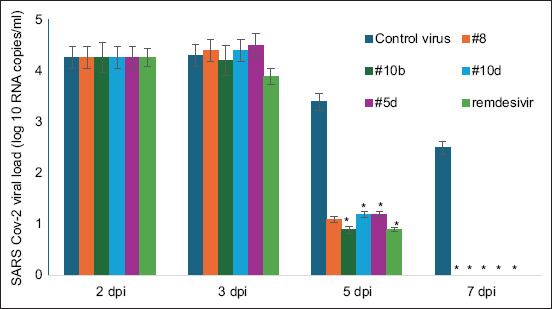
Viral load in oronasal swabs during severe acute respiratory syndrome-related coronavirus infection in Syrian hamsters and under the treatment of heterocycle compounds. *Significant compared with oronasal swabs obtained from animals from virus control group (p < 0.05, p < 0.01).

Hamsters were infected with the SARS-CoV-2 delta variant by intranasal and oral inhalation at a dose of 106 TCD50/mL. The virus peaked in the oronasal swabs at 2 dpi with a concentration of 4.2 log10 RNA copies/mL (Figure-4). Oronasal probes were collected for SARS-CoV-2 detection at 3, 5, 7, and 9 dpi. Swabs were taken from all animals’ orals and noses before administering the virus at 3, 5, 7, and 9 dpi. Samples from the nose and mouth mucous membranes were tested for the virus’s presence. Hamsters had productive viral infections after 48 h of being infected. In the control group, a decrease in viral load was observed after 120 h of infection, but all hamsters continued to test positive. 5^th^-day oronasal swab viral loads in treated animals were 2 log10 lower than in untreated animals. In hamsters from the virus control group, positive probes for SARS-CoV-2 remained detectable after day 7, whereas in treated animals, they disappeared before day 7.

### Changes in blood cell populations

Decreases in mature lymphocytes, monocytes, and neutrophils, along with increases in early forms, were observed in hamster peripheral blood during delta strain SARS-CoV-2 infection [24]. We will present data on both the therapeutic and preventive effects, as they yield near identical results. The viral infection’s pathological manifestations are almost completely eliminated by the therapeutic and prophylactic effects of drugs, as shown in Table-2.

**Table-2:** Blood cells populations at 7 dpi under the therapeutic action of heterocycle compounds (%).

Cells	Control	SARS Cov-2 7 dpi	Preparatin #5d 7 dpi	Preparatin #8 7 dpi	Preparatin #10b 7 dpi	Preparatin #10d 7 dpi
Lymphoblast	0.3 ± 0.1	2.0 ± 0.3	0.9 ± 0.2	1.1 ± 0.1	4.0 ± 0.3	0.9 ± 0.1
Lymphocyte	61.8 ± 7.1	30.9 ± 4.2	65 ± 9.3	77.2 ± 8.1	42.3 ± 6.7	58 ± 6.2
Limph.patol.	-	1.0 ± 0.1	-	-	-	-
Monoblast	0.4 ± 0.1	1.0 ± 0.1	-	2.9 ± 0.5		1.0 ± 0.1
Monocyte	3.9 ± 0.4	4.0 ± 0.5	2.1 ± 0.3	1.2 ± 0.1	3.9 ± 0.9	
Myeloid	-	2.0 ± 0.4	1.1 ± 0.1	-	1 ± 0.05	-
Metamyelocyte	1.4 ± 0.1	3.3 ± 0.5	2 ± 0.5	2.0 ± 0.6	1.0 ± 0.1	3.1 ± 0.4
Band	17.5 ± 2.6	30.2 ± 2.8	19 ± 4.8	10.0 ± 2.7	27 ± 8.6	20.7 ± 7.3
Segment	13.3 ± 2.1	22.5 ± 1.9	8.8	4	20	13
Pathol neutrophil	-	3.0 ± 0.9	-	-	2.8 ± 0.7	0.9 ± 0.1
Eosinophil	1.4 ± 0.2	0.1 ± 0.1	0.1 ± 0.05	0.5 ± 0.1	0.1 ± 0.01	0.5 ± 0.01
Basophil	0.01 ± 0.01	0.01 ± 0.01	0.01	-	-	0.01 ± 0.005
Destroyed	-	0.01 ± 0.01	1.0	1.9 ± 0.3	0.9 ± 0.02	-

SARS Cov-2=Severe acute respiratory syndrome-related coronavirus, dpi=day post-infection

### Changes in bone marrow cell populations

**Figure-5:**
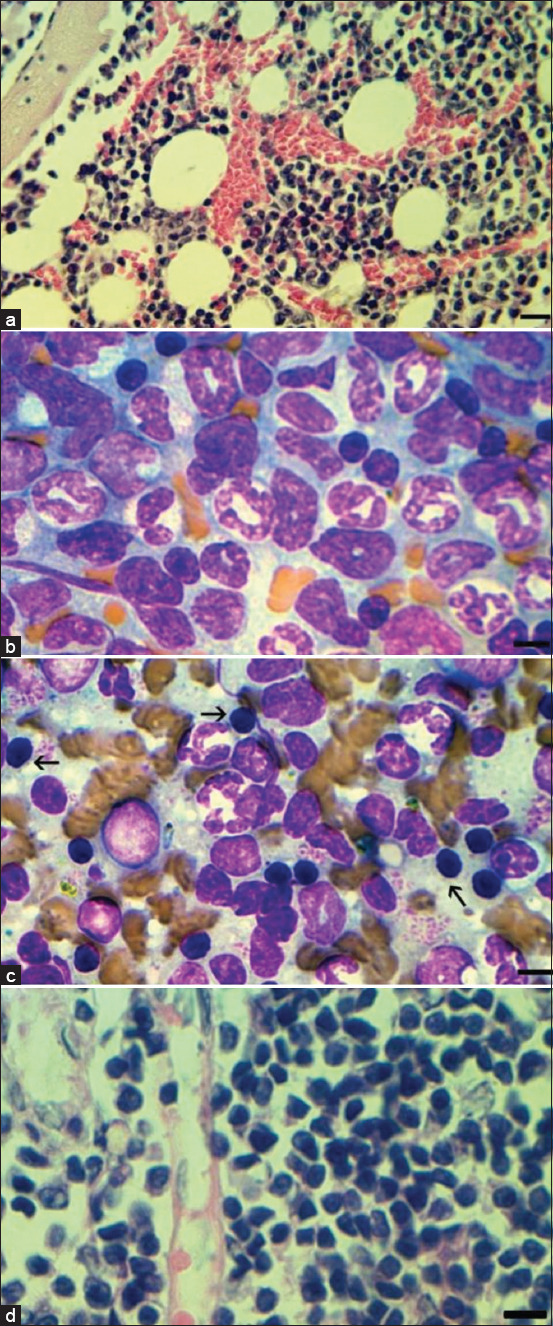
Changes in bone marrow and spleen in severe acute respiratory syndrome-related coronavirus infection under the therapeutic action of heterocycle compounds. (a) BM section (9^th^-day post-infection [dpi]) preparation #5d. Staining hematoxylin and eosin (H&E), preserved structure and cellularity; scale bar 50 μm. Preserved structure of tissue. (b) BM smear (18^th^ dpi). restored erythroid population; preparation #5d; scale bar 10 μm. (c) Spleen smear (18^th^ dpi) proliferation of late erythroblasts (arrowed); preparation #10d; scale bar 10 μm. (d) LN section (9^th^ dpi) preparation #8. Staining H&E, preserved structure and cellularity; scale bar 10 μm.

The number of nucleated erythroid cells in bone marrow significantly decreases upon SARS-CoV-2 infection. While the count of other cells decreased, the number of lymphoid cells, monoblasts, and monocytes increased. Myeloid cells and metamyelocytes decreased while neutrophil content increased within the myeloid population. The delta variant of SARS-CoV-2 results in the development of abnormal lymphocytes and neutrophils [28]. In this section, for clarity, we will focus on the single drug (5d) and its effect on myeloid populations, as other drugs exhibit comparable therapeutic effects. 9^th^-day drug administration does not obstruct the similar bone marrow changes seen in the positive control group (Figure-5a). A more rapid recovery of all cell populations was observed on the 18^th^ day compared to the positive control (Table-3 and Figure-5b). Histological examination showed a transient increase in erythroid and myeloid populations in the spleen and lymph nodes, which was rapidly reversed (Figure-5c and d).

**Table-3:** Myelogram at 9 and 18 dpi under the therapeutic action of heterocycle compounds (%).

Cells	Control	SARS Cov-2 9 dpi	SARS Cov-2 18 dpi	Preparatin #5d 9 dpi	Preparatin #5d 18 dpi
Proerythroblast	2.09 ± 0.5	2.27 ± 0.7	1.85 ± 0.4	3.1 ± 0.5	4,1 ± 0.6
Basophil erythroblast	9.97 ± 1.1	5.02 ± 0.8	6.92 ± 1.0	4.6 ± 0.9	2,7 ± 0.8
Polychromatophil erythroblast	15.53 ± 2.4	6.01 ± 0.8	10.25 ± 1.3	5.9 ± 2.1	17.9 ± 2.3*
Ortochrom erythroblast	9.11 ± 0.9	6.36 ± 1.5	7.21 ± 1.1	8.8 ± 0.9	11,2 ± 3.2**
Lymphoblast	1.07 ± 0.2	3.98 ± 0.7	2.01 ± 0.5	3.5 ± 0.6	2.3 ± 0.5
Lymphocyte	3.31 ± 0.7	11.09 ± 1.1	4.97 ± 0.7	8.9 ± 1.2	5.2 ± 0.8
Limph.patol.	-	0.45 ± 0.06	0.29 ± 0.04	1.1 ± 0.4	0.1 ± 0.04*
Monoblast	0.81 ± 0.1	3.09 ± 0.9	0.88 ± 0.07	2.9 ± 0.7	1.0 ± 0.02
Monocyte	1.17 ± 0.3	4.57 ± 1.1	2.36 ± 0.5	3.8 ± 0.8	1.5 ± 0.4
Myeloid	13.65 ± 2.9	4.18 ± 0.9	5.76 ± 0.9	3.9 ± 0.5	14.3 ± 2.7
Metamyelocyte	25.62 ± 4.7	11.32 ± 2.5	20.34 ± 4.4	5.2 ± 1.0	20.1 ± 4.3
Band	14.13 ± 2.1	32.64 ± 6.4	27.55 ± 5.8	26.1 ± 4.4	15.6 ± 3.1*
Segment	2.35 ± 0.6	4.18 ± 1.2	7.52 ± 2.0	3.7 ± 0.5	3.3 ± 0.3*
Pathol neutrophil	-	0.81 ± 0.1	0.53 ± 0.06	2.1 ± 0.5	0.1 ± 0.01*
Eosinophil	0.91 ± 0.2	1.45 ± 0.09	1.02 ± 0.2	3.2 ± 0.4	0.2 ± 0.01
Basophil	0.08 ± 0.01	0.09 ± 0.01	0.02 ± 0.01	0.1 ± 0.04	0.01 ± 0.01
Destroyed	0.01 ± 0.01	1.80 ± 0.3	0.24 ± 0.02	3.2 ± 0.3	0.1 ± 0.01
Macroph. Resident	0.14 ± 0.05	0.24 ± 0.05	0.09 ± 0.01	1.6 ± 0.05	0.1 ± 0.01
Macroph. Island	0.02 ± 0.01	0.09 ± 0.01	0.07 ± 0.01	1.6 ± 0.03	0.1 ± 0.01
Megakaryoblast	0.01 ± 0.01	0.06 ± 0.01	0.07 ± 0.01	1.6 ± 0.02	0.03 ± 0.02
Megakaryocyte basophil	0.01 ± 0.01	0.05 ± 0.01	0.02 ± 0.01	1.6 ± 0.1	0.01 ± 0.01
Megakaryocyte azurophil	0.01 ± 0.01	0.04 ± 0.01	0.02 ± 0.01	1.6 ± 0.1	0.01 ± 0.01
Mitos	0.01 ± 0.01	0.18 ± 0.01	0.01 ± 0.01	1.6 ± 0.02	0.01 ± 0.01

* Significant, compared to corresponded positive control (p < 0.05).

** Tendency, compared to corresponded positive control (p < 0.1). SARS-Cov-2=Severe acute respiratory syndrome-related coronavirus. dpi=day post-infection

### Inner organs histopathology

**Figure-6:**
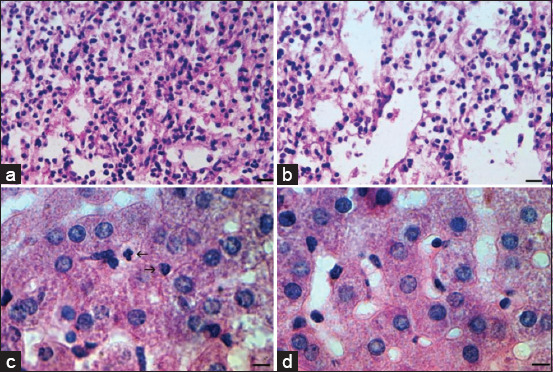
Lung and liver pathology in hamsters after infection with severe acute respiratory syndrome-related coronavirus (delta strain) 5-day post-infection and under the therapeutic action of heterocycle compounds analogs. (a) Diffuse lymphocytic-macrophage infiltrates. Hematoxylin and eosin (H&E) staining; scale bar 50 μm. (b) Mild pathological manifestations under the therapeutic action of preparation #8. (c) Light neutrophilic diapedesis in the liver. (d) Absence of neutrophils in liver tissues under the therapeutic action of preparation #10b. H&E staining; scale bar 10 μm.

The lack of significant histopathological changes in the internal organs (lungs, liver, and kidneys) was observed under therapeutic exposure to SARS-CoV-2 virus analogs. The alterations vanished by 14-day post-implantation. In Figure-6a, diffuse lymphocytic-macrophage infiltrates are observed in SARS-Cov-2 lung path. The symptoms were moderated by exposure to all the investigated drugs, as shown in Figure-6b. In Figure-6c, liver pathology due to SARS-Cov-2 is predominantly manifested by leukocyte diapedesis. After exposure to all the drugs, this pathology was no longer present (Figure-6d).

These new heterocycle compounds have been demonstrated to induce antiviral effects against various picornaviruses, as shown in Figures S1–S4. Some of the studied compounds exhibit strong antiviral activity against SARS-CoV-2 both *in vitro* and *in vivo*. The single introduction of these derivatives into an animal induces an antiviral state that lasts for at least 48 h.

**Figure-S1:**
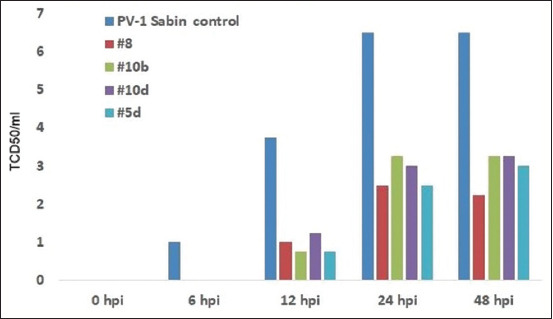
The suppresion of poliovirus-1 (Sabin) replication in Hep-2 cells by prophylactic treatment by heterocycle compounds.

**Figure-S2:**
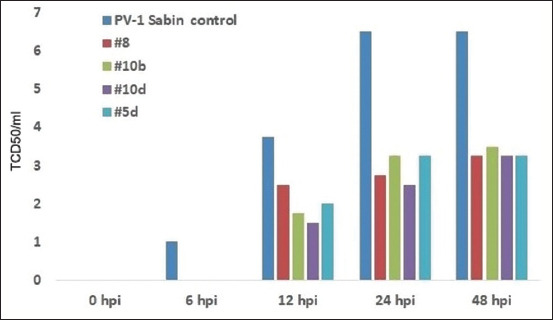
The suppresion of poliovirus-1 (Sabin) replication in Hep-2 cells by therapeutic treatment by heterocycle compounds.

**Figure-S3:**
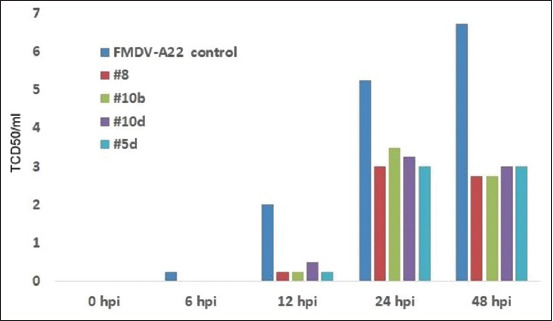
The suppresion of foot and mouth desease virus (A-22) replication in BHK-21 cells by prophylactic treatment by heterocycle compounds.

**Figure-S4:**
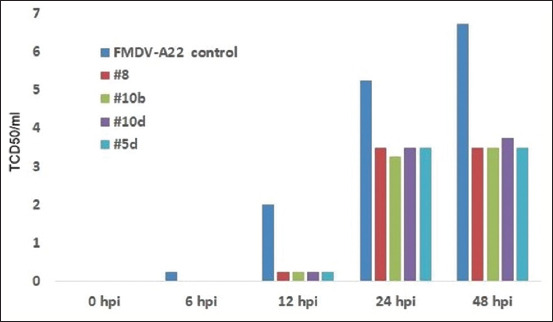
The suppresion of foot and mouth desease virus (A-22) replication in BHK-21 cells by therapeutic treatment by heterocycle compounds.

## Discussion

While vaccines have proven effective against certain viral infections, finding vaccines for other viral diseases is a challenging and costly endeavor [26, 27]. The urgent demand exists for creating efficient antiviral medications and adapting established drugs to new applications. The goal was to generate novel pyrimidine and pyridazine derivatives during the synthesis process. To produce novel derivatives of pyrimidine and pyridazine in order to synthesize future anomalous nucleosides using them as a basis 4 heterocycle compounds were chosen for further antiviral study following initial experiments on ten selected compounds. *In vitro* and *in vivo*, the nucleobase analogs from the original novel significantly inhibited SARS-Cov-2. Most modern antiviral drugs are derived from abnormal nucleosides, which function as antimetabolites of natural DNA and RNA building blocks. Research into the synthesis of nucleoside analogs began in the 1950s. These nitrogen-containing organic compounds, extensively studied for their antiviral activity, are demonstrated to be highly effective drugs for treating a range of viral illnesses [24].

Nucleoside analogs behave as false metabolites, binding to and inhibiting viral nucleic acid polymerases. Nucleoside analogs halt viral replication by being incorporated into DNA or RNA chains, resulting in termination or mutation accumulation due to error catastrophe. *In vitro* and *in vivo* studies have documented these phenomena in a range of RNA viruses [25–27]. Our proposed substances exhibit antiviral activity against SARS-CoV-2 that is comparable to known antivirals. Abnormal nucleosides impede SARS-CoV-2 by obstructing its RNA-dependent RNA polymerase [27, 29, 30] or inducing mutations in novel viral RNA strands [8, 28, 30]. Thymine replacements were predominantly identified among mutations in SARS-CoV-2, according to available data [28, 31].

Our study reveals that analogs of NB inhibit various RNA viruses, including poliovirus (Note: Data for poliovirus is not provided within the text). Newly synthesized heterocycle compound analogs inhibited SARS-CoV-2 replication significantly, leading to a decrease in viral RNA load in the supernatant under *in vitro* conditions using delta and omicron strains of SARS-CoV-2 virus. Heterocycle compound analogs suppressed SARS-CoV-2 replication *in vitro* and *in vivo*, as shown by improved pathological manifestations in hamsters’ blood, bone marrow, and internal organs [22].

Our experimental infection of Syrian hamsters with SARS-Cov-2 resulted in a significant decrease in pathogenic symptoms and viral levels, as shown through both *in vivo* and *in vitro* experiments.

## Conclusion

Using delta and omicron strains of SARS-CoV-2 virus, newly created heterocycle compound analogs dramatically reduced SARS-CoV-2 multiplication, resulting in a drop in viral RNA load in the supernatant under *in vitro* conditions. Improvements in pathological manifestations in the blood, bone marrow, and internal organs of hamsters demonstrated that heterocycle compounds inhibited SARS-CoV-2 replication both in vitro and *in vivo*. Both *in vivo* and *in vitro* tests demonstrating the experimental infection of Syrian hamsters with SARS-Cov-2 revealed a noteworthy reduction in pathogenic symptoms and viral levels.

## Authors’ Contributions

AY, TG, VP, EG, RS, AK, DA, LA, KB, MS, BB, NB, SH, AP, AA, LH, KZ: Methodology and investigation. HGT, VP, EG, S, AP, AA, LH, HA, KZ: Visualization. RS, AK, LA, KZ: Software and data curation. AY, KZ, AP, HA, SH: Writing – original draft, conceptualization, and Writing – review and editing. All authors read and approved the final manuscript.
